# *Houttuynia cordata* Thunb extract modulates G_0_/G_1_ arrest and Fas/CD95-mediated death receptor apoptotic cell death in human lung cancer A549 cells

**DOI:** 10.1186/1423-0127-20-18

**Published:** 2013-03-19

**Authors:** Yuh-Fung Chen, Jai-Sing Yang, Wen-Shin Chang, Shih-Chang Tsai, Shu-Fen Peng, Yuan-Ru Zhou

**Affiliations:** 1 Department of Pharmacology, College of Medicine, China Medical University, No 91, Hsueh-Shih Road, Taichung 40402, Taiwan; 2 Department of Biological Science and Technology, College of Life Sciences, China Medical University, No 91, Hsueh-Shih Road, Taichung 40402, Taiwan

**Keywords:** *Houttuynia cordata* Thunb (HCT), G_0_/G_1_ arrest, Apoptosis, Fas/CD95, Lung cancer A549 cells

## Abstract

**Background:**

*Houttuynia cordata* Thunb (HCT) is commonly used in Taiwan and other Asian countries as an anti-inflammatory, antibacterial and antiviral herbal medicine. In this study, we investigated the anti-human lung cancer activity and growth inhibition mechanisms of HCT in human lung cancer A549 cells.

**Results:**

In order to investigate effects of HCT on A549 cells, MTT assay was used to evaluate cell viability. Flow cytometry was employed for cell cycle analysis, DAPI staining, and the Comet assay was used for DNA fragmentation and DNA condensation. Western blot analysis was used to analyze cell cycle and apoptotic related protein levels. HCT induced morphological changes including cell shrinkage and rounding. HCT increased the G_0_/G_1_ and Sub-G_1_ cell (apoptosis) populations and HCT increased DNA fragmentation and DNA condensation as revealed by DAPI staining and the Comet assay. HCT induced activation of caspase-8 and caspase-3. Fas/CD95 protein levels were increased in HCT-treated A549 cells. The G_0_/G_1_ phase and apoptotic related protein levels of cyclin D1, cyclin A, CDK 4 and CDK 2 were decreased, and p27, caspase-8 and caspase-3 were increased in A549 cells after HCT treatment.

**Conclusions:**

The results demonstrated that HCT-induced G_0_/G_1_ phase arrest and Fas/CD95-dependent apoptotic cell death in A549 cells

## Background

In Taiwan, 26 individuals per 100,000 died from lung cancer each year, based on reports from the “People Health Bureau of Taiwan”. Surgery, radiotherapy, and chemo-therapy are used for treating lung cancer patients [1-3]. However, those treatments are not satisfactory. Induction of cell cycle arrest and/or apoptosis in lung cancer cells has been considered an influential treatment strategy [4-6]. Many researchers have focused on selectively killing cancer cells or reducing cell number through the induction of cell cycle arrest and apoptosis [6,7].

Morphological changes in apoptotic cells include cell membrane blebbing, DNA or chromatin condensation, and caspase activation [8-10]. Previous studies have demonstrated that the cell membrane death receptor played an important role in apoptosis [11,12]. Death receptor signaling is mediated through FasL and Fas/CD95 receptor protein interaction followed by activation of caspase-8 [13-16]. The activation of caspase-3 by caspase-8 is responsible for the cleavage of cellular substrates [13-20]. Cleavage of cellular substrates degrades the chromosomes into fragments during apoptosis [13-16,21].

*Hottuynia cordata* Thunb (HCT), also called E-Sung-Cho is a Chinese herb used to treat several different diseases (e.g., bovine mastitis, influenza etc.) [22-24]. In addition, HCT has value in treating allergic inflammation [25,26], viral infections and anaphylaxis [27-29]. Many studies reported HCT extract has anti-leukemia [30,31] and anti-colon cancer activity [32,33]. HCT inhibits the growth of HER2/neu-overexpressing breast cancer cells [34]. In this study, we determined if HCT would have anti-human lung cancer activity and if such effects would be associated with inhibition of cell growth in the human lung cancer line A549.

## Methods

### Preparation of HCT

Ethanol extract of *Houttuynia cordata* Thunb (yield: 6.73% of dry wt.) was obtained by 48 h incubation at room temperature. The ethanol extract was filtered through a 0.45 μm filter (Osmonics, Minnetonka, MN, USA), lyophilized and kept at 4°C. The dried extract was re-solubilized in PBS before use as previously described [32,33].

### Chemicals and reagents

RPMI-1640 cell culture medium (Gibco BRL, Life Technologies, MD, USA), DAPI (4,6-diamidino-2-phenylindole dihydrochloride), low-melting agarose, MTT (3-(4,5-dimethylthiazol-2-yl)-2,5-diphenyltetrazolium bromide), and DMSO (dimethyl sulfoxide) were purchased from Sigma (St. Louis, MO, USA). FBS (Fetal bovine serum), penicillin/streptomycin, PI (propidium iodide) and trypsin-EDTA were obtained from Life Technologies (Carlsbad, CA, USA). Proteinase K was purchased from Roche Diagnostics Gmbh (Mannheim, Germany). Ac-DEVE-pNA and Ac-IETD-pNA were purchased from R&D Systems Inc., (MN, USA). All other chemicals used were of analytical grade.

### Cell culture

Human lung cancer A549 cells were obtained from the Bioresource Collection and Research Center (BCRC, Hsinchu, Taiwan), originally from the American Type Culture Collection (ATCC, USA). Cells were maintained in RPMI-1640 containing 100 mL/L FBS with 100,000 U/L penicillin and 100 mg/L streptomycin.

### Cell viability

A549 cells were plated onto 96-well plates and incubated with HCT (0, 125, 250 and 500 μg/ml) for 24 and 48 h. MTT was added to each well then incubated for an additional 4 h at 37°C. The blue formazan product was dissolved in 100 μL of DMSO. The plates were read at O.D.570 nm using a spectrophotometric plate reader (Bio-Rad, Tokyo, Japan). The experiments were performed in triplicate (n = 3). Cell viability was calculated as O.D. of drug-treated sample/O.D. of none treated sample × 100% as previously described [32,35].

### Cell cycle transition and apoptosis determination

For cell cycle and apoptosis determination, A549 cells were plated onto 24-well plates and incubated with HCT (0, 125, 250 and 500 μg/ml) for 24 h. Cells were fixed gently in 70% ethanol at 4°C and then re-suspended in phosphate-buffered saline (PBS) containing 40 μg/ml PI, 0.1 mg/ml RNase and 0.1% Triton X-100 for 30 min at 37°C. Cell cycle transition and apoptosis were then analyzed by flow cytometry (FACS Calibur™; Becton Dickinson, NJ, USA) as previously described [35].

### DAPI staining

A549 cells were plated onto 24-well plates and treated with HCT (0 and 500 μg/ml) for 24 h. After HCT treatment, cells were fixed in 4% paraformaldehyde and then incubated with 1 μg/ml of DAPI staining solution in darkness. The apoptotic cells were observed by fluorescence microscopy (Zeiss, Oberköchen, Germany) as previously described [32,35].

### Comet assay

A549 cells were plated onto 24-well plates and incubated with HCT (0 and 500 μg/ml) for 24 h, 1 × 10^4^ cells were mixed with 150 μL 0.75% low-melting agarose held at 37°C and layered onto a pre-treated slide with 1.5% regular agarose. After agarose were solidified on a chilled plate, the slides were transferred to the same lysis buffer, held at room temperature for 4 h and stained with propidium iodide as previously described [36].

### Western blotting

A549 cells were plated onto T-75 flasks and treated with HCT (0, 125, 250 and 500 μg/ml) for 24 h. Total cell lysates were prepared as previously described. Thirty μg of total protein applied to SDS-PAGE and transferred onto a polyvinylidene fluoride membrane (PVDF; Millipore). After blocking, the blots were incubated with the appropriate dilution of specific monoclonal antibodies for cyclin D1, cyclin A, CDK 4, CDK 2, p27, caspase-8 and caspase-3 (Santa Cruz Biotechnology, USA). Blots were washed and then incubated with horseradish peroxidase-conjugated secondary antibody (Santa Cruz Biotechnology, USA). Protein expressions were detected using enhanced chemiluminescence kits (Amersham, ECL Kits) as previously described [37].

### Caspase-8 and caspase-3 activities assays

A549 cells were plated onto T-75 flasks and incubated with HCT (0, 125, 250 and 500 μg/ml) for 24 h. A549 cells were collected in a protein lysis buffer (50 mM Tris–HCl, 1 mM EDTA, 10 mM EGTA, 10 mM digitonin and 2 mM DTT). Cell lysates were centrifuged at 15,000 × g at 4°C and then incubated with caspase-3 and caspase-8 specific substrates (Ac-DEVE-pNA and Ac-IETD-pNA) with reaction buffer in a 96-well plate at 37°C for 1 h. Caspase activity was determined by measuring O.D. 405 nm of the released pN using a spectrophotometric plate reader (Bio-Rad, Tokyo, Japan) as previously described. The experiments were performed in triplicate [37].

### Immunostaining assay for Fas/CD95 protein levels

Fas/CD95 cell surface antigen expression was measured by flow cytometry. A549 cells were plated onto 24 well and treated with HCT (0, 125, 250 and 500 μg/ml) for 12 h. Cells were collected and rinsed in PBS. Fas/CD95 was analyzed by direct immune-fluorescence staining. FITC-conjugated anti-Fas/CD95 and its FITC- conjugated isotype mAb (BD Biosciences Pharmingen, San Diego, CA, USA) were analyzed using a flow cytometer as previously described [38].

### Statistical analysis

The experiments were performed in triplicate (n = 3) and all data were expressed as the mean ± standard error. Student’s *t*-test was used for single variable comparison. For multiple variable comparisons, data were analyzed by one-way ANOVA followed by Dunnett’s test. *P* < 0.05 was considered significant.

## Results

### Effect of HCT on viability of A549 cells

**Figure 1:**
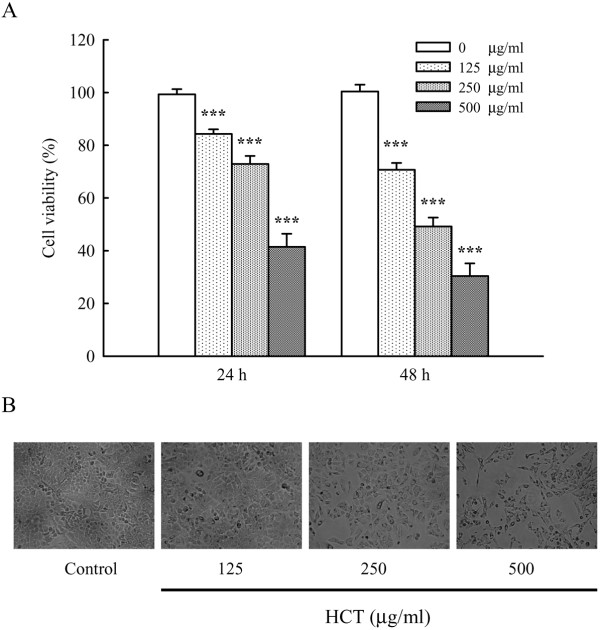
**Effects of HCT on cell viability and morphological changes in human lung cancer A549 cells.** (**A**) The A549 cells were treated with HCT (0, 125, 250 and 500 μg/ml) for 24 and 48 h, the viable cells were determined by MTT assay as described in Materials and Methods. The experiments were performed in triplicate (n = 3). ***p < 0.001 was considered significantly different in comparison with the control. (**B**) The cells’ morphological changes were examined and photographed by phase-contrast microscopy.

Cell viability of HCT (0, 125, 250 and 500 μg/ml) treated A549 cells was determined by MTT assay for 24 and 48 h. As shown in Figure 1A, HCT reduced cell viability in a concentration- and time-dependent manner (***p < 0.001, compared to HCT 0 μg/ml group). The inhibition of HCT in A549 viability was 15.73%, 27.13%, and 58.57% for 125, 250, and 500 μg/ml at 24 h; 29.32%, 50.81%, and 69.60% for 125, 250, and 500 μg/ml at 48 h, respectively. HCT also induced morphological changes seen as cell shrinkage and rounding (Figure 1B).

### Effect of HCT on cell-cycle distribution and apoptosis in A549 cells

**Figure 2:**
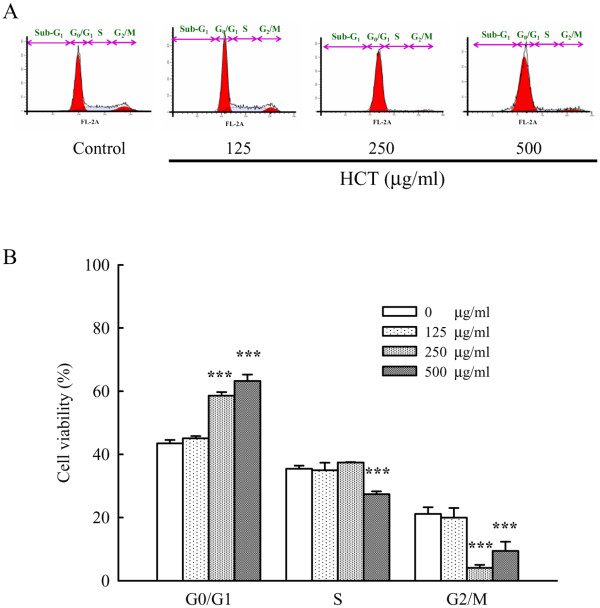
**Effects of HCT on cell-cycle transition in human lung cancer A549 cells.** (**A**) The A549 cells were treated with HCT (0, 125, 250 and 500 μg/ml) for 24 h, and then were harvested for determination the distribution of cell cycle by flow cytometry and (**B**) quantitative results were expressed as described in Materials and Methods. The experiments were performed in triplicate (n = 3). Data represents mean ± S.D. of three experiments. ***p < 0.001 was considered significantly different in comparison with the control.

**Figure 3:**
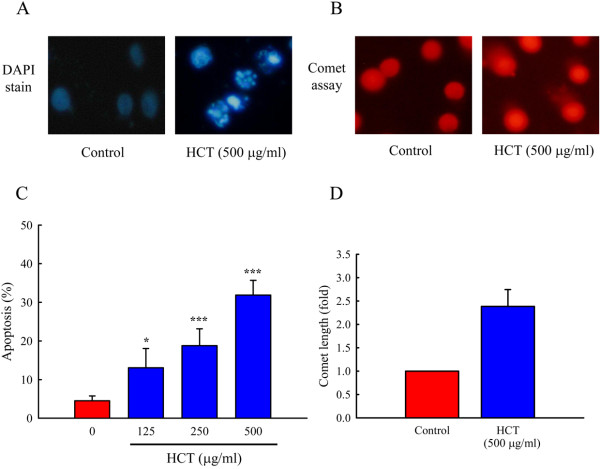
**Effects of HCT on DNA condensation, damage and apoptosis in human lung cancer A549 cells.** (**A**) A549 cells stained with DAPI to observe DNA condensation after 24 h of treatment with 500 μg/ml of HCT. (**B**) A549 cells were examined DNA damage by Comet assay after 24 h of treatment with 500 μg/ml of HCT. Cells were photographed under fluoresce microscopy (x200) as described in Materials and Methods. (**C**) A549 cells were treated with HCT (0, 125, 250 and 500 μg/ml) for 24 h, and then were harvested for determination the sub-G1 (apoptosis) cell population by flow cytometry as described in Materials and Methods. (**D**) The bar diagram of the length of Comet tail. The experiments were performed in triplicate (n = 3). *p < 0.05, ***p < 0.001 was considered significantly different in comparison with the control.

To evaluate the effects of HCT on cell-cycle distribution and apoptosis, A549 cells were treated with HCT (0, 125, 250 and 500 μg/ml). Figure 2A and B showed that HCT caused an arrest of cell-cycle transition in the G_0_/G_1_ phase, and the proportion of cells in the G_0_/G_1_ phase increased markedly in a concentration-dependent manner. As shown in Figure 2B, the cell cycle distribution of G_0_/G_1_ by HCT (0, 125, 250 and 500 μg/ml) treatment was 43.48%, 45.06%, 58.59%, and 63.25%, respectively. In addition, A549 cells treated with HCT (0, 125, 250 and 500 μg/ml) showed a significant increase in the sub-G1 apoptotic cell population, and the apoptotic cell population was 4.53%, 13.07%, 18.77% and 31.89%, respectively (Figure 3C). These results indicated HCT inhibited cell growth.

### Effects of HCT on G_0_/G_1_ phase-associated protein levels

**Figure 4:**
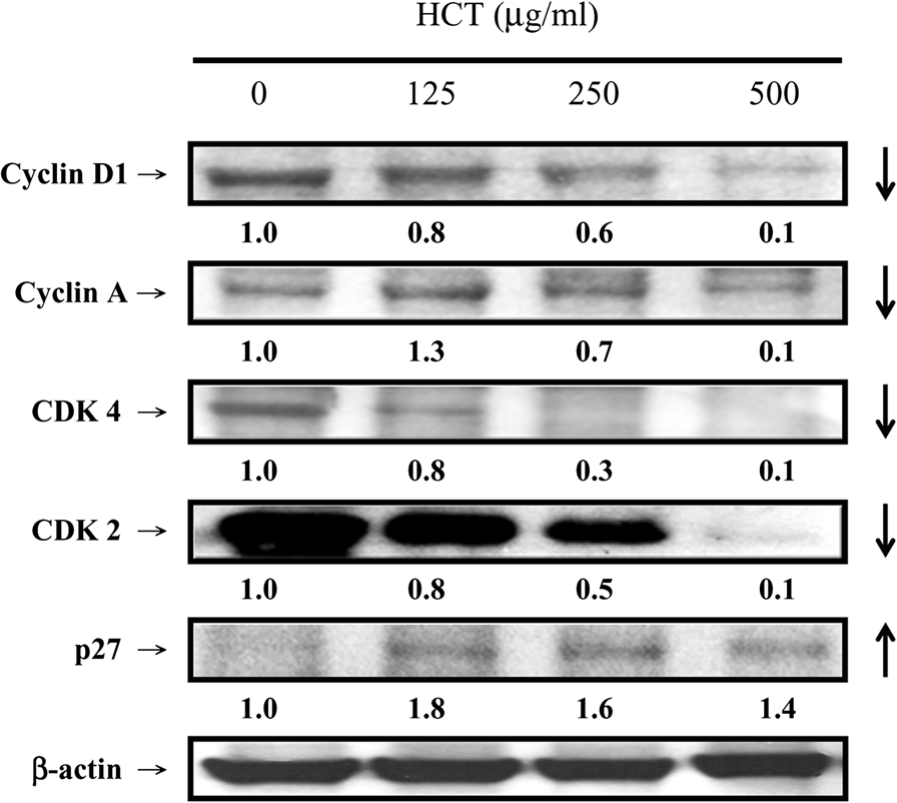
**Effects of HCT on G**_**0**_**/G**_**1 **_**relative protein levels in A549 cells.** A549 cells were treated with HCT (0, 125, 250 and 500 μg/ml) for 24 h and then performed western blotting analysis for Cyclin D1, Cyclin A, CDK4, CDK2, and p27 in HCT treated cells as described in Materials and Methods. The experiments were performed in triplicate (n = 3).

We investigated the protein levels of the G_0_/G_1_ phase. Figure 4 showed that HCT caused an increase in the protein level of p27 and decreased protein levels of CDK4, CDK2, Cyclin D1 and Cyclin A in A549 cells. HCT (0, 125, 250 and 500 μg/ml) treatment increased p27 expression ratio by 1, 1.8, 1.6, and 1.4, respectively. Among the protein levels of G_0_/G_1_ phase, decrease of CDK4 was the most significant one. HCT decreased protein levels of CDK4 by 0%, 20%, 70%, and 90%, respectively.

### Effect of HCT on DNA condensation and damage in A549 cells

Effects of HCT on nuclear morphological change were examined by DAPI staining and DNA damage by the Comet assay. As shown in Figure 3A and B, cells exhibited nuclear shrinkage and DNA condensation after a 24 h-incubation with HCT (500 μg/ml) (Figure 3A). Cells exposed to HCT had an increase in DNA damage as seen in the Comet assay (Figure 3B), and the length of Comet tail was 2.39 fold increase (Figure 3D). The results suggest that HCT induces morphological changes and DNA damage in A549 cells.

### Effects of HCT on caspase-8 and caspase-3 activities

**Figure 5:**
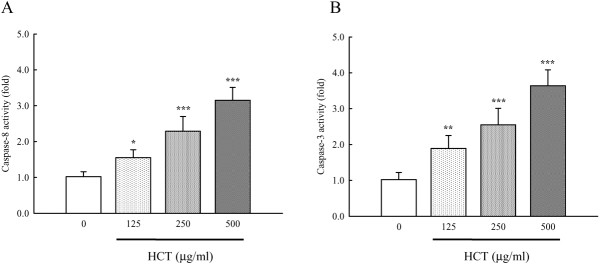
**Effects of HCT on caspases-8 and caspase-3 activities in human lung cancer A549 cells.** The A549 cells were treated with HCT (0, 125, 250 and 500 μg/ml) for 24 h, and then total cell extracts were incubated with (**A**) caspases-8 specific substrate (Ac-IETD-pNA) and (**B**) caspase-3 specific substrate (Ac-DEVE-pNA) respectively. The release of pNA was measured at 405 nm by a spectrophotometer as described in Materials and Methods. The experiments were done in triplicate (n = 3). *p < 0.05, **p < 0.01, ***p < 0.001 was considered significantly different in comparison with the control.

**Figure 6:**
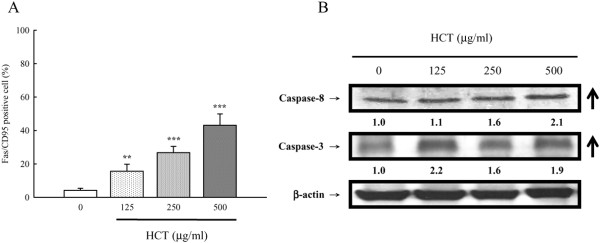
**Effects of HCT on A549 cells in the Fas/CD95-dependent apoptotic pathway.** The A549 cells were treated with HCT (0, 125, 250 and 500 μg/ml) for 12 h, and (**A**) Fas/CD95 protein expression levels was detected by immune staining and analyzed by flow cytometry as described in Materials and Methods. The experiments were performed in triplicate (n = 3). **p < 0.01, ***p < 0.001 was considered significantly different in comparison with the control. (**B**) Caspase-8 and caspase-3 protein expression levels in HCT-examined cells were analyzed by Western blotting as described in Materials and Methods.

**Figure 7:**
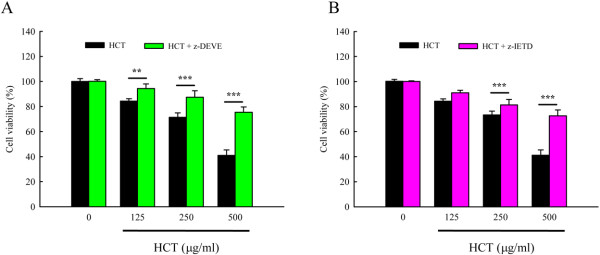
**Effects of caspases-3 and caspase-8 specific inhibitor on cell viability in HCT- treated human lung cancer A549 cells.** Cells were pretreated with (**A**) the caspase-3 inhibitor (z-DEVE-fmk) and (**B**) the caspase-8 inhibitor (z-IETD-fmk) for 1 h after exposure to HCT (0, 125, 250 and 500 μg/ml) for 24 h exposure, viable cells were determined by MTT assay as described in Materials and Methods. The experiments were performed in triplicate (n = 3). The experiments were performed in triplicate. **p < 0.01, ***p < 0.001 was considered significantly different in comparison with the control.

We determined if HCT would stimulate the caspase-9, caspase-8 and caspase-3 activity in A549 cells. Figure 5, panel A and panel B, show that HCT increased caspase-8 and caspase-3 activities in a concentration-dependent manner but caspase-9 activity was unaffected (data not shown). HCT (125, 250 and 500 μg/ml) treatment increased caspase-8 activity by 51.96%, 124.51%, and 208.82%, respectively; increased caspase-3 activity by 85.29%, 150%, and 256.86%, respectively. Our results showed that HCT-induced apoptosis was mediated through the activation of caspase-8 and caspase-3. Thus, we determined whether the Fas/CD95 (death receptor protein) contributes to HCT-induced apoptosis. Figure 6, panel A showed that Fas/CD95 protein level was increased in a concentration-dependent manner in A549 cells. Figure 6, panel B also showed that caspase-8 and caspase-3 protein levels increased in a concentration-dependent manner when A549 cells were treated with HCT. HCT (125, 250 and 500 μg/ml) treatment increased caspase-8 protein expression by 1.1, 1.6, and 2.1 fold, respectively; increased caspase-3 expression ratio by 2.2, 1.6, and 1.9 fold, respectively. To further verify the involvement of caspase-8 and caspase-3 in HCT-induced apoptosis, cells were pretreated with the caspase-8 inhibitor (z-IETD-fmk) and the caspase-3 inhibitor (z-DEVE-fmk). It can be seen in Figure 7, panels A and B showed that the caspase-3 and caspase-8 inhibitors inhibited the apoptotic effects of HCT.

## Discussion

The death receptor apoptotic pathway has previously been proposed as an anti-cancer drug target in human lung cancer [39,40]. Traditional Chinese medicine (TCM) that could stimulate the death receptor apoptotic pathway should have therapeutic potential in human lung cancer treatment [41-43]. HCT has been used as TCM in Taiwan for many years [44]. The pharmacological activities of HCT include immuno-regulatory, anti-inflammatory, anti-micro bacterial, anti-viral, and anti-cancer effects [24,25,27-32]. HCT was reported to be active against leukemia, colorectal cancer and HER2/neu-overexpressing breast cancer cells [30-34]. However, the anti-lung cancer effects have not been well-studied. We previously reported that 450 μg/ml of HCT had anti-cancer activity in human primary colorectal cancer cells from patients [33] and HT29 human colon adenocarcinoma cells [32]. In the present study, HCT had anti-lung cancer activity (Figure 1), and this activity was concentration-, and time-dependent. HCT contains quercetin and quercetin 3-β-D-glucoside (isoquercitrin) which have anti-viral activity [28,45]. Chou SC, *et. al.* indicated that quercitrin and isoquercitrin isolated from methanolic extracts of *Houttuynia cordata* showed excellent DPPH radical scavenging activities with IC50 values of 31 μM and 63 μm, respectively [46]. Also, HCT contains chlorogenic acid [47] which has anti-leukemia effects. In this study, we suggested that ethanolic extract of HCT have anti-lung cancer activity.

Previous studies have shown that HCT induces human lymphoblastic leukemic Molt-4 cell death through an endoplasmic reticulum stress pathway [31]. Banjerdpongchai R, *et. al.* suggested that ethanolic extract of HCT induces human leukemic HL-60 and Molt-4 cell apoptosis through a mitochondrial apoptotic pathway [30].

Evidences showing the link between apoptosis and the cell cycle are compelling. Regulation of cell cycle traverse involves activations of cyclin-dependent kinases (CDKs). CDKs (e.g. CDK1, CDK2, CDK4/6) is paired with the cyclins (e.g. cyclins A, B, E, D_1-3_) [48]. Activation of CDKs by cyclins leads to phosphorylation of the retinoblastoma protein (pRb) [49] and leads to diminished binding of pRb to the E2F transcription factor, which various genes necessary for cell cycle progression. The activities of CDKs are antagonized by CDK inhibitors. CDK inhibitors directly interact with CDKs and negatively regulate the activity of CDKs. p27^kip1^, belongs to the Cip/Kip (kinase inhibitor protein) family, inhibits most CDKs [50]. Dysregulation of cell cycle is one of the most potent stimuli for apoptosis induction [51]. p27^kip1^[52], cyclin D1 [53], has been shown to influence the apoptotic process. Direct inhibition of CDKs, induction of endogenous CDK inhibitors (e.g. p27^kip1^) or down-regulation of cyclins required for CDK activation (e.g. cyclin D_1_) are mechanisms of inhibition of cell cycle progression [48]. Our results showed that HCT increased p27 expression, decreased cyclin D1, cyclin A, CDK 4, and CDK 2 which leaded to cycle G_0_/G_1_ arrest. HCT induced apoptosis through caspase8/caspase-3 activation (Figure 5A,B) and up-regulated the protein levels of Fas/CD95 (Figure 6A), caspase-8 and caspase-3 (Figure 6B), while HCT did not affect the caspase-9 activity (data not shown) in A549 cells. In addition, HCT induced growth inhibition was significantly attenuated by the specific caspase-3 and caspase-8 inhibitors (Figure 7A, B). One interpretation of our findings is that HCT induces A549 cell apoptosis and activates caspase-8, and −3 through the Fas/CD95-mediated death receptor apoptotic pathway.

## Conclusions

**Figure 8:**
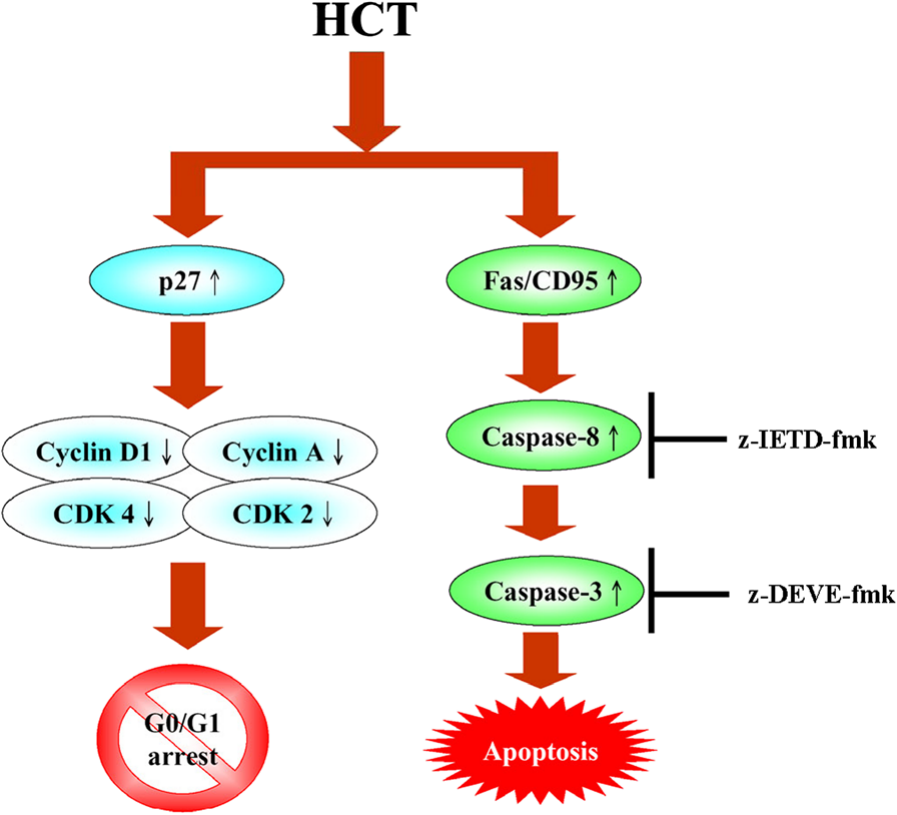
**A proposed model of HCT modulates G**_**0**_**/G**_**1 **_**arrest and Fas/CD95- mediated death receptor apoptotic cell death on human lung cancer A549 cells.**

In conclusion, this study demonstrated that HCT has anti-lung cancer activity by modulating G0/G1 arrest and stimulating the Fas/CD95 protein level, which leads to caspase-8 and caspase-3 activation resulting in the induction of apoptosis in human lung cancer A549 cells (Figure 8).

## Abbreviations

HCT: *Houttuynia Cordata* Thunb; DAPI: 4,6-Diamidino-2-Phenylindole Dihydrochloride; MTT: 3-(4,5-dimethylthiazol-2-yl)-2,5-diphenyltetrazolium bromide; FBS: Fetal Bovine Serum; PBS: Phosphate-Buffered Saline; DMSO: Dimethyl Sulfoxide; PI: Propidium Iodide; PVDF: Polyvinylidene Fluoride Membrane.

## Competing interests

The authors declare that they have no competing interests related to this work.

## Authors’ contributions

YFC conceived for the study, and participated in its design, performed the statistical analysis and figure drawing, coordination, draft and revise the manuscript. JSY helped to draw the figures and drafted the manuscript. WSC carried out the cell viability, cell cycle transition, G_0_/G_1_ relative protein levels assay and caspase activities assay. SCT helped to draft the manuscript. SFP carried out the DAPI staining and Comet assay. YRZ performed immunostaining assay for Fas/CD95 protein levels. All authors read and approved the final manuscript.
